# Oxidation of crotyl alcohol by N-chloro-4-methylbenzene sulphonamide in acidic medium and in alkaline media in the presence of os(VIII) catalyst-a kinetic pathway

**DOI:** 10.3906/kim-2003-67

**Published:** 2020-08-18

**Authors:** Priyamvada SHARMA, Riya SAILANI, Anita MEENA, Chandra Lata KHANDELWAL

**Affiliations:** 1 Department of Chemistry, University of Rajasthan, Jaipur India; 2 Department of Chemistry, JNV University, Jodhpur India

**Keywords:** Electron transfer reaction, reaction kinetics, mechanism, osmium(VIII) catalysis, crotyl alcohol, chloramine-T

## Abstract

The kinetic pathway of oxidation of crotyl alcohol by sodium salt of *N* -chloro-4-methylbenzene sulphonamide (chloramine-T) in acidic and alkaline medium has been studied. The speciation of chloramine-T has been made to suggest a proper and reasonable reaction mechanism. The thermodynamic quantities such as activation energy and activation entropy are evaluated in acidic as well as in catalysed alkaline medium. An anticipated reaction mechanism has been suggested.

## 1. Introduction

Chloramine-T is a sodium salt of *N* -chloro-4-methylbenzene sulphonamide and this reagent is used as an important antiseptic and disinfectant in chemical analysis [1]. This reagent is not only an important oxidising agent but has also been employed as a chlorinating agent [2]. Apart from this, chloramine-T is considered to be an important analytical reagent in both chemical and pharmaceutical analysis [3] more particularly drugs and syrups.

The unique feature of this reagent is its amide nitrogen being in anion form (negative). Thus the chlorine bound to sulphonamide is positively charged and is, therefore responsible both for oxidation and chlorination [4].

The chemistry of chloramine-T from the view point of kinetics has been extensively studied [5–8] still there are gaps such as the speciation of its species both in acid [9–11] and alkaline [12–14] media which are not yet well defined. Moreover, the reactions of chloramine-T in alkaline medium are comparatively slow and it is this reason that platinum group metal ions [15,16] were considered effective catalysts in this medium. In most, kinetic studies of oxidation of unsaturated alcohols have been studied in the absence [17] and presence of heterogeneous catalysts [18,19]. Transition metal ions such as Ru(III) [20], Ag(I) [21], Os(VIII) [22], Pd(III) [23] were also taken as homogenous catalysts. But still the product of reactions between unsaturated alcohols and metal ions in solution phase is such an enigma.

As far as the details of oxidation of these alcohols are concerned, a very less amount of information is reported about its kinetic pattern of oxidation of crotyl alcohol by *N* -chloro-4-methylbenzene sulphonamide. Since these unsaturated alcohols have been extensively employed in solution with various types of oxidants both metal and nonmetal, yet the details related to the kinetic mechanistic pathway of the reactions apart from identification of oxidation products and dependence of the rate on concentration of hydrogen ion are known to a lesser extent. In view of these observations the title study was undertaken to gain more about the redox chemistry of this important reagent.

## 2. Results and discussion

### 2.1. Results

**Figure 1:**
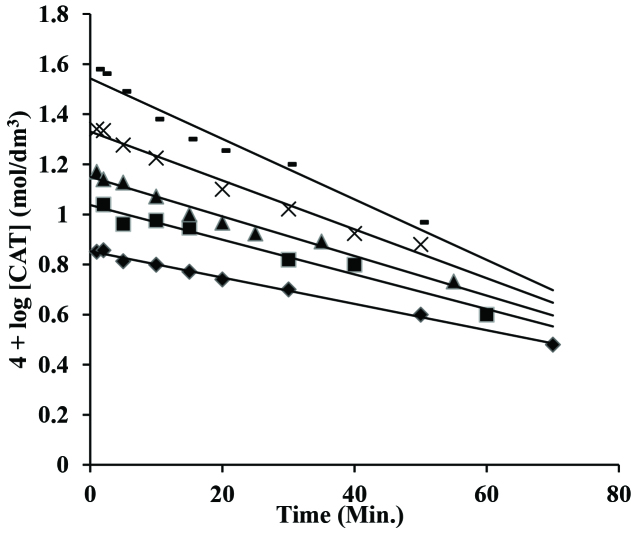
Pseudo I order plots in oxidation of crotyl alcohol and CAT in acidic medium. [CA] = 5 ×10^-2^ mol/dm^3^; [HClO_4_] = 5×10^-2^ mol/dm^3^ [CAT] = (1) ♦ 1.0 ×10^-3^; (2) ■1.5 ×10^-3^; (3) ▲ 2.0 ×10^-3^; (4) ×3.0 ×10^-3^; (5) 5.0 ×10^-3^ mol/dm^3^ and 35 C

**Part-I: oxidation of crotyl alcohol by chloramine-T in acid aqueous medium** The chloramine-T (CAT) concentration was ranged from 1 ×10^-3^ to 5 ×10^-3^ mol/dm^3^ while the concentrations of other components of reaction were kept constant at [CA] = 2 ×10^-2^ mol/dm^3^; [H^+^] = 5 ×10^-2^ mol/dm^3^ at 35 ±0.2 C. Initial rates (k_i_) were analysed by plain mirror method and a graph between initial rate constant (k_i_) and the oxidant concentration were made that produced a linear line which crossing the origin conformed first order with respect to the CAT. However, pseudo I order graphs were also done (Figure 1) by applying crotyl alcohol and [H^+^] to be 5 ×10^-2^ mol/dm^3^ at 35 ±0.2 C respectively. Pseudo I order rate constants (k’, s^-1^) were independent of oxidant concentrations which was further conforming first order dependence with respect to chloramine-T.

The crotyl alcohol concentration (henceforth written as CA) was ranged from 2.0 ×10^-3^ to 1.0 ×10^-2^ mol/dm^3^ while the concentrations of other components of reaction were kept constant at [CAT] = 2 ×10^-3^ mol/dm^3^ and [H^+^] = 5 ×10^-2^ mol/dm^3^ at 35 ±0.2 C. The rate is found to be independent of crotyl alcohol concentration.

P -toluene sulphonamide concentration was ranged from 2 ×10^-2^ to 5 ×10^-2^ mol/dm^3^ while theconcentrations of other components of reaction were kept constant at [CAT] = 2 ×10^-3^ mol/dm^3^; [CA] = 5 ×10^-2^ mol/dm^3^; [H^+^] = 5 ×10^-2^ mol/dm^3^ at 35 ±0.2 C. The rate was independent of the p-toluenesulphonamide concentration. This was also confirmed in variation of crotyl alcohol where the order was zerowith respect to crotyl alcohol.

**Figure 2:**
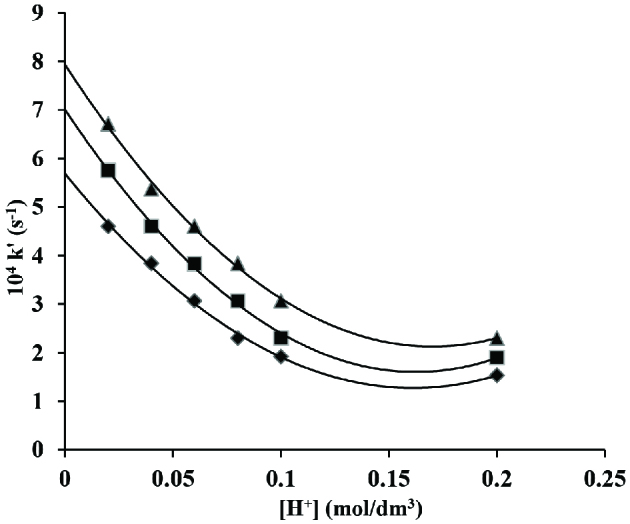
Variation of hydrogen ion in oxidation of crotyl alcohol and CAT in acidic medium. [CAT] = 2 ×10^-2^ mol/dm^3^; [CA] = 5 ×10^-2^ mol/dm^3^; ♦ 35, ■ 40 and ▲ 45 C

Hydrogen cation effect was studied by applying HClO_4_ from 0.02 to 0.2 mol/dm^3^ while the concentrationsof other components of reaction were kept constant at [CAT] = 2 ×10^-3^ mol/dm^3^; [CA] = 5 ×10^-2^ mol/dm^3^ at 35 ±0.2, 40 ±0.2 and 45 ±0.2 C respectively. The rate was inversely proportional to perchloric acid concentration (Figure 2).

The ionic strength effect was inspected by varying LiClO_4_ from 0.1 to 1.0 mol/dm^3^ while the concentrations of other components of reaction were kept constant at [CAT] = 2 ×10^-3^ mol/dm^3^; [CA] = 5 ×10^-2^ mol/dm^3^ and [H^+^] = 5 ×10^-2^ mol/dm^3^ at 35 ±0.2 C. The rate was independent to ionic strength.

The solvent effect on the reaction rate was inspected by employing n-hexane from 5% to 30% (V/V) while the concentrations of other components of reaction were kept constant at [CAT] = 2 ×10^-3^ mol/dm^3^; [CA] = 5 ×10^-2^ mol/dm^3^ and [H^+^] = 5 ×10^-2^ mol/dm^3^ at 35 ±0.2 C. The rate was independent of the *n* -hexane concentration.

The temperature effect on the reaction rate was examined at three temperatures i.e. 35 ±0.2, 40 ±0.2, and 45 ±0.2 C, respectively, while the concentrations of other components of reaction were kept constant at [CAT] = 2 ×10^-3^ mol/dm^3^; [CA] = 5 ×10^-2^ mol/dm^3^ and [H^+^] = 6 ×10^-2^ mol/dm^3^. The thermodynamic constants i.e. activation energy activation entropy were calculated by applying Eyring calculation made between T^1^ versus ln kT^-1^ to be (53.84 ±0.31) kJ/mol and (-139.8 ±1.6) J/K mol, respectively.

Free radical test was done on the reaction with acrylic acid [24]. A longer period even after no white sediment was observed in the reaction. This observation eliminates the probability of a free radical in the reaction. However, it might be possible that as soon as a radical is formed, it immediately interacts with the substrate before diffuse out of the solvent cage to interact with the monomer for polymerization [25].

**Part-II: os(VIII) catalysed oxidation of crotyl alcohol by chloramine-T**

**Figure 3:**
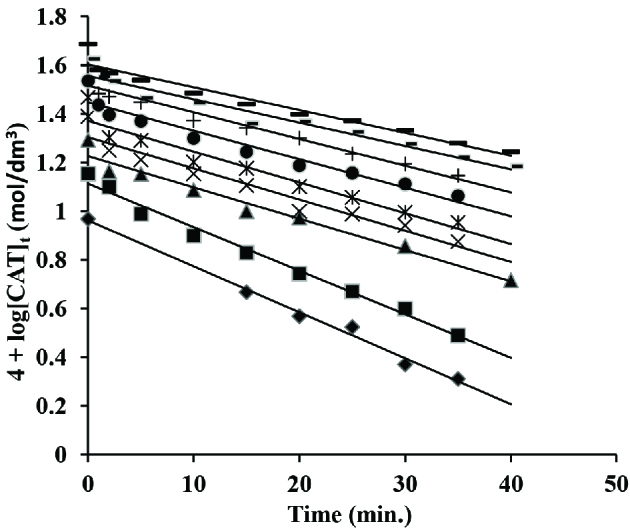
Pseudo I order plots in os(VIII) catalysed reaction in alkaline medium. [CA] = 5 ×10^-2^ mol/dm^3^; [OH^-^] = 1 ×10^-2^ mol/dm^3^; [Os(VIII)] = 5 ×10^-5^ mol/dm^3^ [CAT] = (1) ♦ 1.0 ×10^-3^; (2) ■ 1.5 ×10^-3^ (3) ▲ 2.0 ×10^-3^; (4) ×2.5 ×10^-3^ (5) 3.0 ×10^-3^; (6) • 3.5 ×10^-3^ (7) + 4.0 ×10^-3^; (8) 4.5 ×10^-3^ (9) 5.0 ×10^-3^ mol/dm^3^ and 45 C

The chloramine-T concentration was ranged from 2 ×10^-3^ to 5 ×10^-3^ mol/dm3 while the concentrations of other components of reaction were kept constant at [CA] = 5 ×10^-2^ and 0.1 mol/dm^3^ , [OH^-^] = 1 ×10^-2^ mol/dm ^3 ^ and [Os(VIII)] = 5 ×10^-5^ mol/dm^3^ at 45 ±0.2 C. Since pseudo I order conditions were maintained and thus pseudo I order graphs were made (Figure 3). The pseudo I order rate constants calculated from such graphs were independent of chloramine-T concentrations ensuring first order with respect to chloramine-T.

The crotyl alcohol concentration was ranged from 1×10−3 to 5 ×10−2 mol/dm3 while the concentrations of other components of reaction were kept constant at [CAT] = 2 ×10−3 mol/dm3 ; [Os(VIII)] = 5 ×10−5 mol/dm3 and constant concentrations of [OH−] = 0.005, 0.01 and 0.02 mol/dm3 , respectively at 45 ±0.2 C. Initial rates were found to increase initially and then inclined towards a delimit rate at higher alcohol concentrations. Such a dependence of crotyl alcohol appears to be complex. The dependence of crotyl alcohol can be accounted for the Eq. (1) empirically as follows:

(1)-d[CAT]dt=A[CAT][Crotyl Alcohol]B+C[Crotyl Alcohol]

Where A, B and C are empirical rate constants. Rate become independent of it concentration at 0.04 to 0.1 mol/dm^3^, pseudo I order rate constants obtained in this region are in a good agreement with pseudo I order rate constants obtained in case of variation of concentrations of chloramine-T under pseudo I order conditions (Table).

**Table:** Pseudo I order and second order rate constants in the reaction of crotyl alcohol with chloramine-T in alkaline medium. [OH^-^] = 0.01 mol/dm^3^, Temp. = 45 C

103[CAT] (mol/dm3)	102[CA] (mol/dm3)	105 [Os(VIII)] (mol/dm3)	104 (k’) (s−1)	(k) (mol s/ dm3)
1.0	5.0	5.0	3.454	6.91
1.5	5.0	5.0	3.454	6.91
2.0	5.0	5.0	3.454	6.91
2.5	5.0	5.0	3.454	6.91
3.0	5.0	5.0	3.454	6.91
3.5	5.0	5.0	3.454	6.91
4.0	5.0	5.0	3.454	6.91
4.5	5.0	5.0	3.454	6.91
5.0	5.0	5.0	3.454	6.91
1.0	10	5.0	3.454	6.91
1.5	10	5.0	3.454	6.91
2.0	10	5.0	3.454	6.91
2.5	10	5.0	3.454	6.91
3.0	10	5.0	3.454	6.91
3.5	10	5.0	3.454	6.91
4.0	10	5.0	3.454	6.91
4.5	10	5.0	3.454	6.91
5.0	10	5.0	3.454	6.91
2.0	4.0	5.0	3.454	6.91
2.0	4.5	5.0	3.454	6.91
2.0	5.0	5.0	3.454	6.91
2.0	5.5	5.0	3.454	6.91
2.0	6.0	5.0	3.454	6.91
2.0	6.5	5.0	3.454	6.91
2.0	7.0	5.0	3.454	6.91
2.0	7.5	5.0	3.454	6.91
2.0	8.0	5.0	3.454	6.91
2.0	10.0	5.0	3.454	6.91
2.0	5.0	1.0	0.698	6.91
2.0	5.0	2.0	1.354	6.77
2.0	5.0	3.0	2.0	6.7
2.0	5.0	4.0	2.75	6.90
2.0	5.0	5.0	3.454	6.91
2.0	5.0	6.0	4.12	6.90
2.0	5.0	8.0	5.373	6.7
2.0	5.0	10.0	6.909	6.90

**Figure 4:**
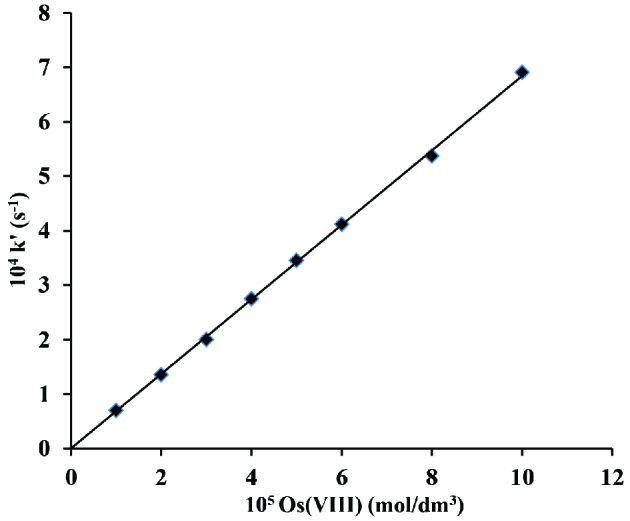
Variation of osmium(VIII) in os(VIII) catalysed reaction in alkaline medium. [CAT] = 2 ×10^-3^ mol/dm^3^; [CA] = 2 ×10^-2^ mol/dm^3^; [OH^-^] = 1 ×10^-2^ mol/dm^3^ and 45 C

The osmium(VIII) concentration was ranged from 1 ×10^-5^ to 10 ×10^-5^ mol/dm^3^ while the concentrations of other components of reaction were kept constant at [CAT] = 2 ×10^-3^ mol/dm^3^; [CA] = 5 ×10^-2^ mol/dm^3^ and [OH^-^] = 1 ×10^-2^ mol/dm^3^ at 45 ±0.2 C. The rate constants graph of pseudo I order were made against the Os(VIII) concentration that produced a linear line which crossing the origin conforms the first order with respect to the catalyst (Figure 4).

**Figure 5:**
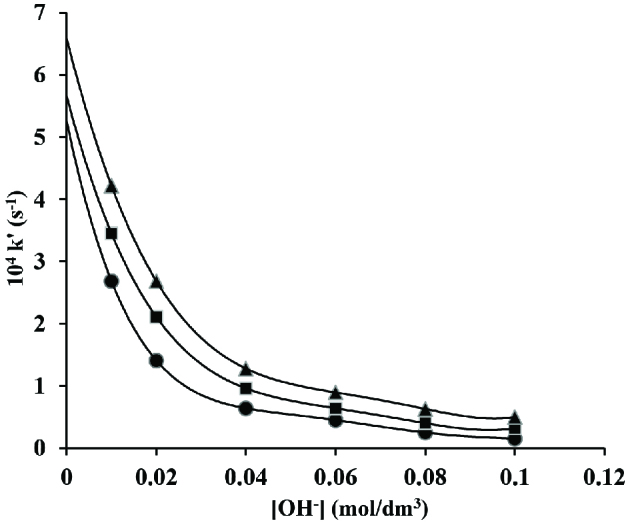
Variation of hydroxide ion in os(VIII) catalysed reaction in alkaline medium. [CAT] = 2 ×10^-3^ mol/dm^3^; [CA] = 5 ×10^-2^ mol/dm^3^; [Os(VIII)] = 5 ×10^-5^ mol/dm^3^; [I] = 1.0 mol/dm^3^; • 40; ■ 45 and ▲ 50 C

The hydroxide ion concentration was ranged from 0.01 to 0.1 mol/dm^3^ while the concentrations of other components of reaction were kept constant at [CAT] = 2 ×10^-3^ mol/dm^3^; [CA] = 5 ×10^-2^ mol/dm^3^; [Os(VIII)] = 5 ×10^-5^ mol/dm^3^ and [I] = 1.0 mol/dm^3^ at 40 ±0.2, 45 ±0.2 and 50 ±0.2 C, respectively. The rate decreases with increasing hydroxide ion concentration (Figure 5).

The ionic strength effect was studied by using NaNO_3_ varying from 0.05 to 1.0 mol/dm^3^ while the concentrations of other components of reaction were kept constant at [CAT] = 2 ×10^-3^ mol/dm^3^; [CA] = 5 ×10^-2^ mol/dm^3^; [Os(VIII)] = 5 ×10^-5^ mol/dm^3^ and [OH^-^] = 1 ×10^-2^ mol/dm^3^ at 45 ±0.2 C. The rate remains unaltered with the sodium nitrate concentration.

The temperature effect on the rate was examined at 40 ±0.2, 45 ±0.2 and 50 ±0.2 C respectively while the concentrations of other components of reaction were kept constant at [CAT] = 2 ×10^-3^ mol/dm^3^; [CA] = 5 ×10^-2^ mol/dm^3^; [OH^-^] = 6 ×10^-2^ mol/dm^3^ and [Os(VIII)] = 5 ×10^-5^ mol/dm^3^. The thermodynamic quantities i.e. activation energy and activation entropy were analysed by using Eyring calculation made between T^-1^ versus ln kT^-1^ to be (54.62 ±0.06) kJ/mol and (-154.19 ±0.31) J/K mol, respectively.

Free radical test was done by adding acrylic acid into the reaction mixture even after a longer period no white sediment was observed in the reaction. This negates the probability of participation of a free radical in the reaction. However, it might be possible that as soon as a radical is formed, it immediately interacts with the substrate before diffusing out of the solvent cage.

### 2.2. Discussion

**Part-I: oxidation of crotyl alcohol by chloramine-T in acid aqueous medium**

The speciation of *N* -chloro-4-methylbenzene sulphonamide known to be chloramine-T is still a question to be settled satisfactorily both in alkaline and acid media. The sodium salt of *N* -chloro-4-methylbenzene sulphonamide being a strong electrolyte reportedly spectates [26,27] in different forms which are governed by Eqs. (2) to (6).

(2)RNClNa⇄Na++RNCl-

(3)H++RNCl-⇄RNHCl

(4)2RNHCI⇄RNCl2+RNH2

(5)H2O+RNHCl⇄HOCl+RNH2

(6)HOCl⇄OCl-+H+

All such species except OCl^-^ are present in acidic medium [28–30]. Since the order is one with respect to chloramine-T (CAT) and rate is not affected by one of the product, namely 4-methylbenzene sulphonamide (RNH_2_), the equilibrium steps (4) and (5) governing RNCl2 and HOCl, respectively, are ruled out. OCl^-^ species conforms to hydroxide ion catalysis which is contrast to observed dependence of hydroxide ion in the title reaction, and thus OCl^-^ reactivity is also ruled out. Interaction of chloramine-T and crotyl alcohol in acid medium, if discussions regarding species of chloramine-T as mentioned above are any guide along with calculations made by Bishop and Jennings on decinormal solutions of chloramine-T, the RNHCl concentration is significant as contrast to that of RNCl^-^ species. Since the order is one with respect to the alcohol and CAT each and rate is decelerated by concentration of hydrogen cation, the subsequent mechanism accounting for such experimental observations can be envisaged.

(7)RNHCl+H+K⇄RNH2+Cl

(8)RNHCl+CA⇄k2k2[Complex]

(9)[Complex]1⇄k3Products

Employing steady state analysis to intermediate [Complex]_1_, the rate law (10) is acquired.

-d[CAT]dt=k1k3K[RNHCl][CA](k2+k3)(1+K[H+])

Since decomposition of the intermediate complex in step (8) is much faster than the rate in the rate determining step (9) i.e. k_2_ »k_3_ , the rate law (10) is reduced to Eq. (11).

11-d[CAT]dt=k1k3K[RNHCl][CA]k2(1+K[H+])

where [RNHCl] and [CA] are the comprehensive concentrations of chloramine-T and alcohol, respectively. Thus, the rate Eq. (11) accounts for all experimental observations successfully. However, RNH^+^_2_Cl is the protonated chloramine-T species which is not similar in view of its already studied involvement in other reactions of chloramine-T reactions in acid medium.

The rate law (11) is further deduced to Eq. (12).

(12)k=-d[CAT]/dt[CA][CAT]=k1k3Kk2.1(1+K[H+])

where ‘k’ is an observed second order rate constant. A graph of k^-1^ versus [H^+^] was made from the Eq. (13) obtained on double reciprocal and then on rearrangement.

1k=k2k1k3(1(K)+[H+])

(13)=k2k1k3K+k2[H+]k1k3

**Figure 6:**
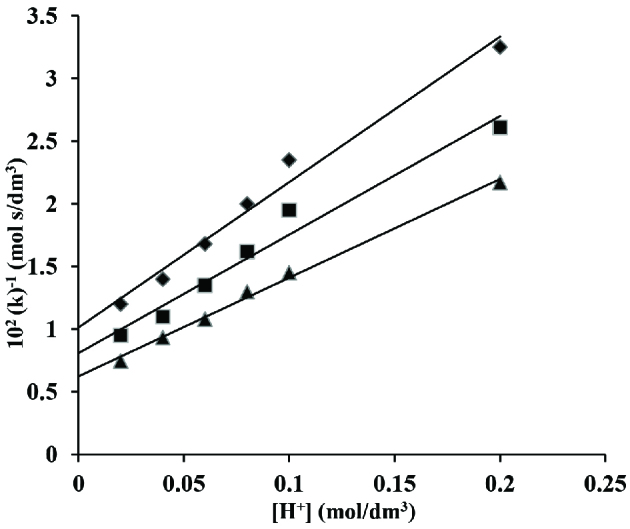
A plot of (k)^-1^ versus [H^+^] in oxidation of crotyl alcohol and CAT in acidic medium. [CAT] = 2 ×10^-2^ mol/dm^3^; [CA] = 5 ×10^-2^ mol/dm^3^; ♦ 35, ■ 40 and ▲ 45 C

Such a plot produced a linear line crossing through origin (Figure 6).

‘K’ from the ratio of slope and intercept was calculated to be 11.44 dm^3^/mol at 35 C, 11.70 dm^3^/mol at 40 C and 12.61 dm^3^/mol at 45 C, respectively. The ratio of ‘k_2_/k_1_’ was calculated from the slope to be 11.59 ×10^-2^, 9.45 ×10^-2^ and 7.87 ×10^-2^ at 35 ±0.2, 40 ±0.2 and 45 ±0.2 C, respectively. So far, as themanner of transfer of electron from the alcohol to the chloramine T is considered, hydrogen anion transfer from α- carbon of alcohol to chloramine-T occurs as represented by the following scheme – 1.

**Scheme 1:**
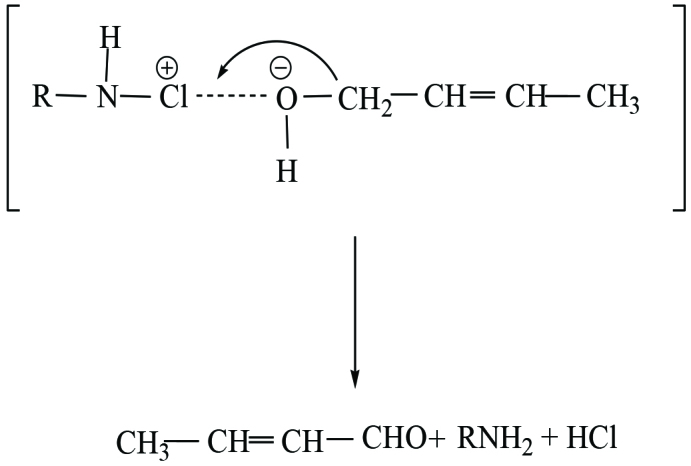


**Part-II: os(VIII) catalysed oxidation of crotyl alcohol by chloramine-T** The solution is red colour of osmium tetraoxide in strongly alkaline medium [31] is reported due to the formation of [OsO_4_ (OH)_2_]^2-^. Such a complex in dilute alkaline medium is converted in to [OsO_3_(OH)_3_]^-^ as reported by Sandell et al. [32]. Since the rate reduces with increasing concentration of hydroxide ion, all the complexes of Os(VIII) following to [OsO_3_(OH)_3_]^-^ species should be less reactive. Further, an sharp reddish brown colour of transient life time appeared on adding crotyl alcohol in Os(VIII) solution at ambient temperature(~5 C) whereas no such colour change was observed with either of these reagents on addition into chloramine-T solution. The formation of chelate between CAT and Os(VIII) has been documented in oxidation of α-hydroxy acids but no such colour change or chelate formation was indicated in the reference reaction.

Therefore, considering experimental considerations, the order is one with respect to [CAT] and [OS(VIII)] each, dependence of complex with respect to crotyl alcohol and inverse hydroxide ion dependence, subsequent mechanism consisting of steps (14) to (18) can be anticipated.

(14)OH-+[OsO3(OH)3]-⇄K1H2O+[OSO4(OH)2]2-

(15)CA+[OsO3(OH)3]-⇄K2[OsO3(OH)3(CA)]-

(16)CA+[OsO4(OH)2]2-⇄K3[OsO4(OH)2(CA)]2-

(17)[OsO3(OH)3(CA)]2-+RNCl-⇄H2Ok1[OsO3(OH)3]2-+>CHO+RNH2+Cl-

(18)[OsO4(OH)2(CA)]2-+RNCl-⇄H2Ok1[OsO3(OH)2]2-+>CHO+RNH2+Cl-

Thus the loss of chloramine-T leads to the rate law (19) or (20).

(19)-d[CAT]dt=[CAT] [Os (VIII)] [CA](k1K2+k2K1K3[OH-]1+K1[OH-]+K2[CA]+K1K3[OH-][CA]

where [CAT] and [Os(VIII)] are the comprehensive concentrations of chloramine-T and osmium(VIII), respectively.

Or

(20)k=[CA](k1K2+k2K1K3[OH-]1+K1[OH-]+K2[CA]+K1K3[OH-][CA]

where ‘k’ is an observed second order rate constant and [CA] is the free equilibrium concentration of crotyl alcohol.

Since limiting rate is not found even at high hydroxide ion concentration, the second term in the numerator is insignificant and can be ignored. This changes the rate law (20) to (21).

(20)k=k1K2[CA]]1+K1[OH-]+K2[CA]+K1K3[OH-][CA]

**Figure 7:**
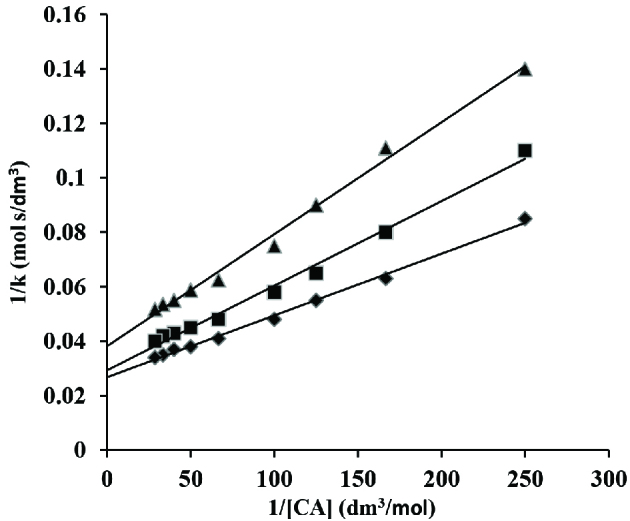
A plot of 1/k versus 1/[CA] in os(VIII) catalysed reaction in alkaline medium. [CAT] = 2 ×10^-2^ mol/dm^3^ ; [Os(VIII)] = 5 ×10^-5^ mol/dm^3^ [OH^-^] = ♦ 5 ×10^-3^; ■ 1 ×10^-2^ ; ▲ 2 ×10^-2^ mol/dm^3^ and 45 C

A graph of 1/k versus 1/[CA] was done from the double reciprocal plot of Eq. (21), a linear line with nonzero intercept was produced (Figure 7).

The calculated slope (S_1_) and intercept (I_1_) from this plot are represented by Eqs. (22) and (23) respectively.

(22)(S1)=1+k1[OH-]k1K2

(23)(I1)=(K2+K1K3[OH-])k1K2

**Figure 8:**
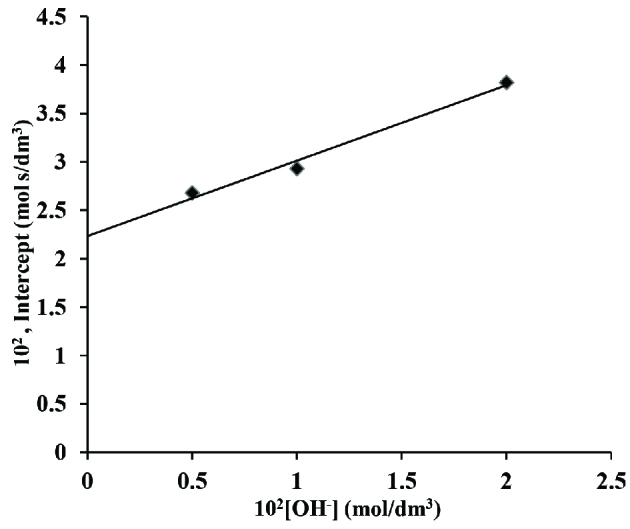
A plot of intercept versus [OH^-^] in os (VIII) catalysed reaction in alkaline medium. Temp. 45 C

**Figure 9:**
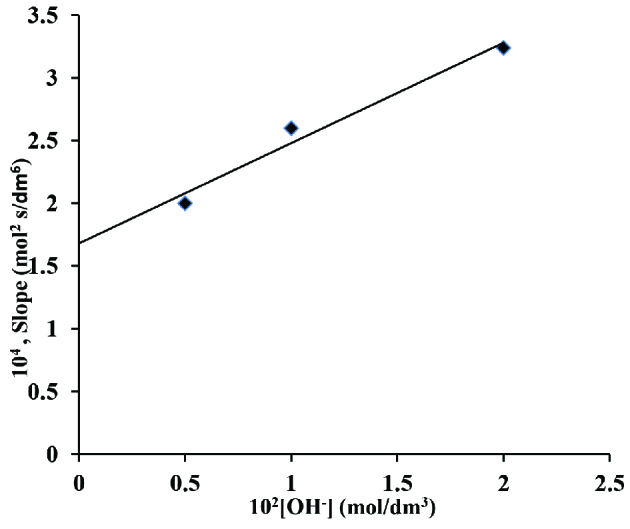
A plot of slope versus [OH^-^] in os (VIII) catalysed reaction in alkaline medium. Temp. 45 C

Further graphs of [OH^-^] versus (I1) and [OH^-^] versus (S_1_) respectively were also made that obtained linear line crossing through origin (Figures 8 and 9), k1 and K1 were calculated from these plots to be 2.24×10^-2^ dm^3^ /mol s^-1^ and 47.6 dm^3^/mol respectively.

**Figure 10:**
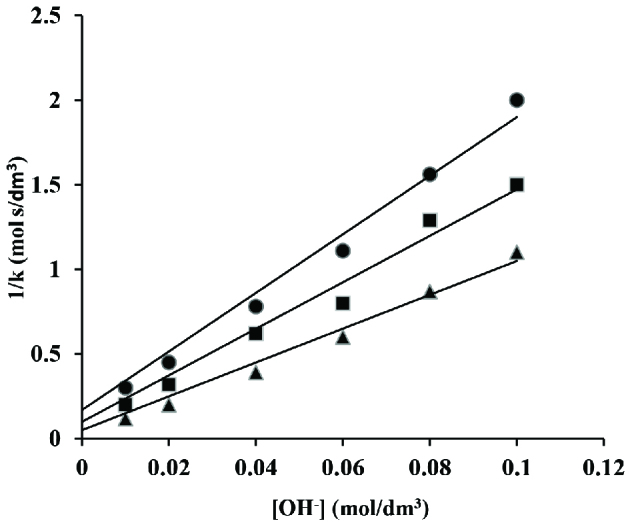
A plot of 1/k versus [OH^-^] in os(VIII) catalysed reaction in alkaline medium. [CAT] = 2 ×10^-3^ mol/dm^3^; [CA] = 5 ×10^-2^ mol/dm^3^; [Os(VIII)] = 5 ×10^-5^ mol/dm^3^; [I] = 1.0 mol/dm^3^; • 40; ■ 45 and ▲ 50 C

Further evaluation of rate constants from Eq. (21) was done by making a graph [OH^-^] versus 1/k at different but constant concentrations of alcohol that also produced linear lines crossing through origin (Figure 10). The intercept (I_2_) and slope (S_2_) evaluated from this graph are given by Eqs. (24) and (25), respectively.

(24)(I2)=1+K2[CA]K1K2[CA]

and

(25)(S2)=K1+K1K3[CA]K1K2[CA]

Since the kinetic analysis of hydroxide ion concentration was limited at only one concentration of crotyl alcohol, this constrained our efforts of graphical analysis for evaluation of K2 and K3 respectively. Nevertheless, these values were calculated by rearranging Eqs. (24) and (25), respectively. Further, reemploying earlier determined values of k1 and K1 in rearranged Eqs, K_2_ and K_3_ respectively were calculated.

From Eq. (24)

(I2)k1=1K2[CA]+

or

[(I2)k1-1]=1K2[CA]

or

(26)K2=1(I2)(k1-1)[CA]

similarly from Eq. (25)

(S2)=K1k1K2[CA]+K1K3k1K2

(26)=K1(I2-1k1)+K1K3k1K2

The value of ‘K_3_’ was determined from this Eq. (26) which was found to be 17.06 ×10^2^.

The manner of transfer of electron from the alcohol to the oxidant in catalysed oxidation by Os(VIII) can be represented by scheme – 2.

A complex ion is formed between Os(VIII) species and crotyl alcohol in which hydrogen anion transfer from the α-carbon atom of alcohol reduces the complex which on attack by a molecule of chloramine-T releases the catalyst and a process of two electrons are consummated on reduction of chloramine-T.

## 3. Experimental

### 3.1. Materials and method

Sodium salt of *N* -chloro-4-methylbenzene sulphonamide (chloramine-T) was procured (98%, E. Merck Ltd, Mumbai, Maharashtra, India) and was in practice as received. Since the salt is highly soluble in water, an aqueous solution of the reagent (~0.1 mol/dm^-3^) is sufficiently stable provided it is kept in dark coloured bottles at refrigerated temperature (~5 C). The solution of chloramine-T was standardized iodometrically [33,34]. All other reagents were of analytical grade and were employed as supplied. Double distilled water was used in the study which was distilled from alkaline permanganate.

### 3.2. Kinetic procedure

Reaction mixtures were clutched in glass stoppered erlenmeyer flasks which were painted dark from the outside, these flasks were immersed in an isothermal water bath at ±0.1 C. The reactions were normally initiated by adding temperature preequilibrated solution of chloramine-T and the initiation time was estimated when fifty percent of the contents from the pipette were discharged into reaction mixture. An exact fraction (5 cm^3^) of the reaction mixture was taken out at regular intervals and then assessed iodometrically.

Initial rates (k_i_, mol/dm^3^ s^-1^) were reckoned by applying plain mirror method [35]. Pseudo I order and second order graphs were also made for comparable concentrations of the reactants and wherever reaction conditions permitted. The rates in triplicate were duplicable to within ±2%.

### 3.3. Stoichiometry

The reaction stoichiometry was calculated by employing surplus amount of chloramine-T over that of substrate under experimental kinetic conditions. This reaction was remained to take place in isothermal water bath more than 6 ca h after conforming of the reaction completion (test of the alcohol was negative). Excess chloramine-T was analysed iodometrically, the results show that a mole of substrate entails a mole of oxidant in the reaction as shown by Eq. (28).

(27)CH3-CH=CH-CH2-OH+RNHCl→CH3-CH=CH2-CHO+RNH2+HCl

where R stands for CH_3_C_6_H_4_SO_2-_.

The reduction product of chloramine-T as *p* -toluene sulphonamide was later on confirmed qualitatively [26].

### 3.4. Product analysis

The oxidation product of crotyl alcohol was identified as crotonaldehyde and the product derivative hydrazone was confirmed with the help of chromatography, IR, NMR and mass spectroscopy. The product of crotyl alcohol also gives the silver mirror with Tollen’s reagent (ammonical silver nitrate).

The characterization of the product was made by thin layer chromatography (TLC). Thin layer chromatograms were conducted on Merck silica gel G plates in ethyl acetate: chloroform (8:2) and in the column chromatographic fractionations silica gel (60–120 mesh) used. Spots on TLC plates were visualized by spraying with 2% cerric ammonium sulphate in 2N H2 SO4 or with iodine vapours.

**Figure 11:**
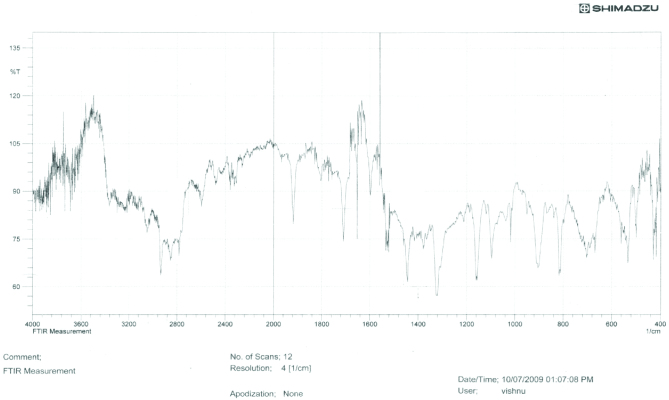
IR spectra of crotonaldehyde.

Such a product was then characterized by employing various instrumental techniques such as follows:

**IR spectral analysis** The IR spectrum (Figure 11) showed absorption band at 2720–2820 cm^-1^ C-H stretching frequency of aldehydic group due to Fermi resonance. The absorption band at 1705 cm^-1^ indicates the presence of C=O of carbonyl group. The absorption band at 2950 cm^-1^ indicates the C-H stretching frequency. The band at 1456 cm^-1^ indicates the C-H asymmetric bending vibrations and at 1367 cm^-1^ C-H symmetry bending vibration of CH_3_ group.

**Figure 12:**
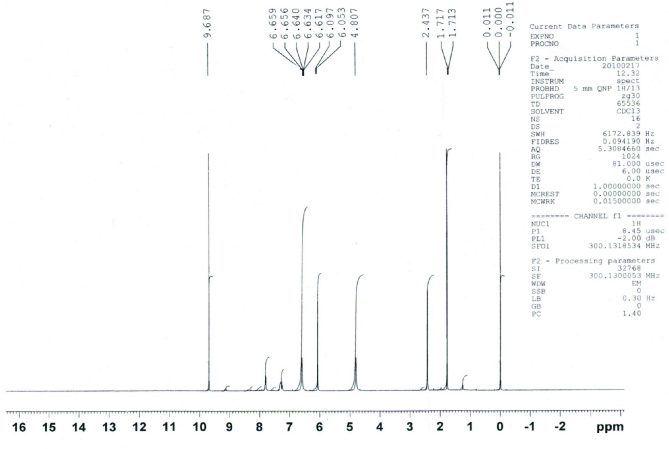
^1^ H NMR spectra of crotonaldehyde.

**^1^H NMR analysis** ^1^H NMR spectrum was also obtained in CDCl_3_ employing 300MHz spectrometer using TMS as reference (Figure 12). In ^1^ H NMR spectrum signal at δ 9.68 ppm is due to aldehydic group. A doublet at δ 6.05 ppm is due to =C-H proton adjacent to aldehydic group. A quintet at δ 6.65 ppm is due to =C-H proton adjacent to methyl group. A doublet at δ 1.71 ppm is due to methyl group of crotonaldehyde.

**2, 4-dinitrophenylhydrazone derivative** Method of preparation: suspend 0.25 g of 2,4-dinitrophenylh ydrazine in 5 ml of methanol and add 0.5 mL of concentrated sulphuric acid cautiously. Warm the solution. Filter the warm solution if necessary and add a solution of 2 mL crotonaldehyde in 1 mL of methanol. Filter the derivative formed and recrystallise from methanol or ethanol. Melting point 190 °C was recorded in soft glass capillaries in an electrothermal melting point apparatus.

**Figure 13:**
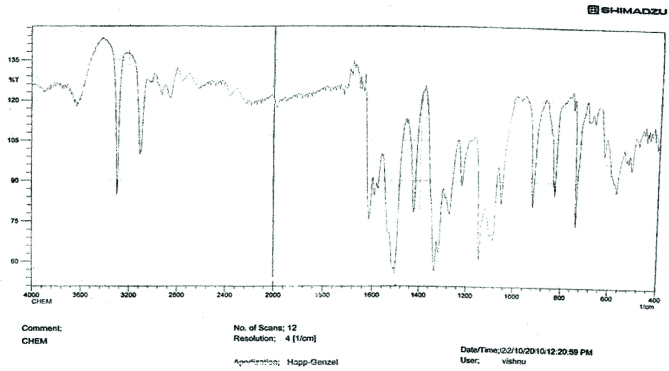
IR spectra of 2,4-dinitrophenylhydrazone.

**Figure 14:**
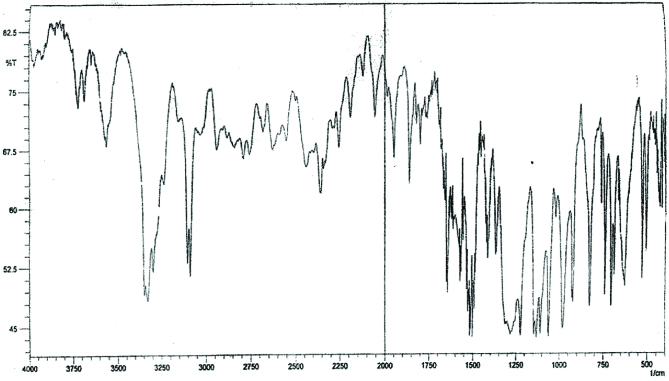
IR spectra of 2,4-dinitrophenylhydrazine.

**IR spectral analysis of 2, 4-dinitrophenylhydrazone** The important peaks observed in the infrared spectrum were at 3300 cm^-1^ indicates the presence of N-H stretching frequency of a secondary amine (Figure 13). Whereas in spectrum of 2,4-dinitrophenyl hydrazine this peak is in doublet form showing N-H stretching frequency of primary amine (Figure 14). The absorption band at 1500 (N-O stretching), 1150 (N=C stretching), 1450 and 1360 cm^-1^ (-CH_2_ bending) were observed.

**Figure 15:**
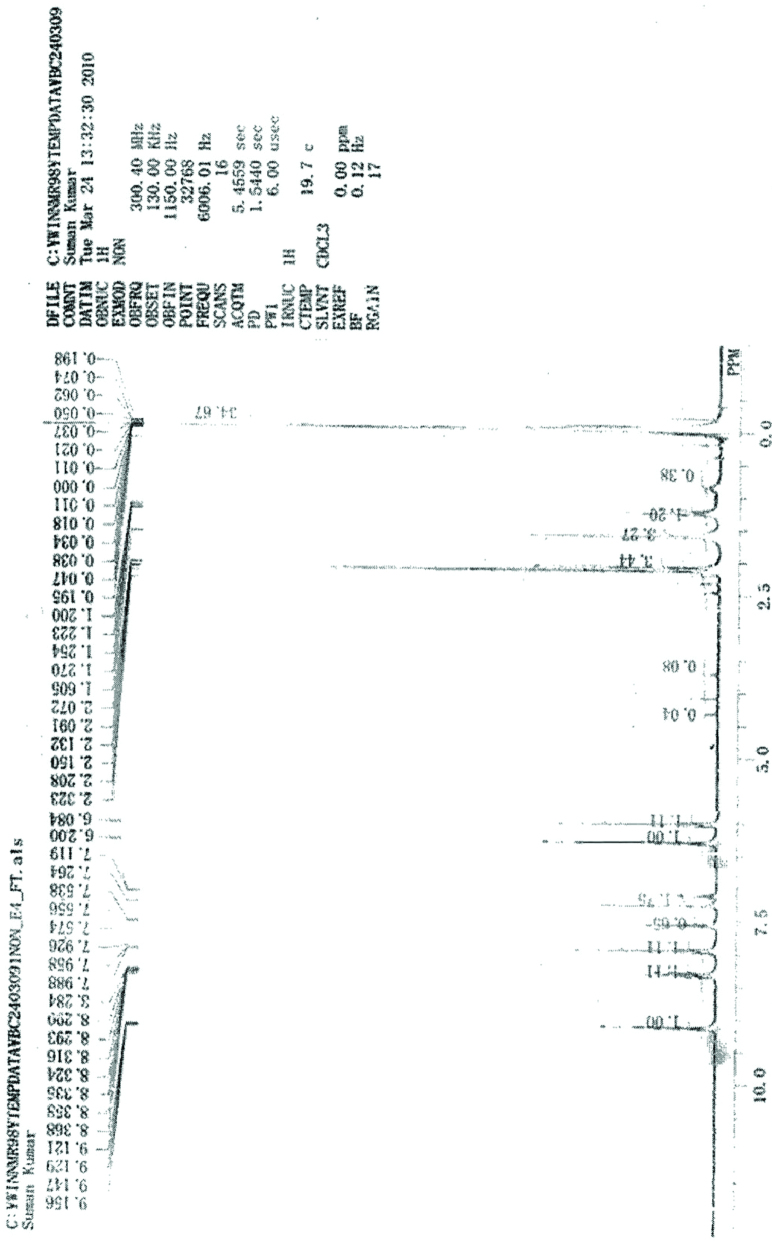
^1^H NMR spectra of 2,4-dinitrophenylhydrazone.

**^1^H NMR analysis of 2, 4-dinitrophenylhydrazone** ^1^ H NMR spectrum was obtained by dissolving hydrazone derivative of product in CDCl_3_ using TMS as reference (Figure 15). In ^1^H NMR spectrum proton of –NH was indicated at δ 6.0 ppm as a singlet. A singlet at δ 6.2ppm was obtained for CH- proton adjacent to NH- group. A multiplet was obtained in the range δ 7.0–9.0 ppm that attributes to the presence of aromatic protons.

**Figure 16:**
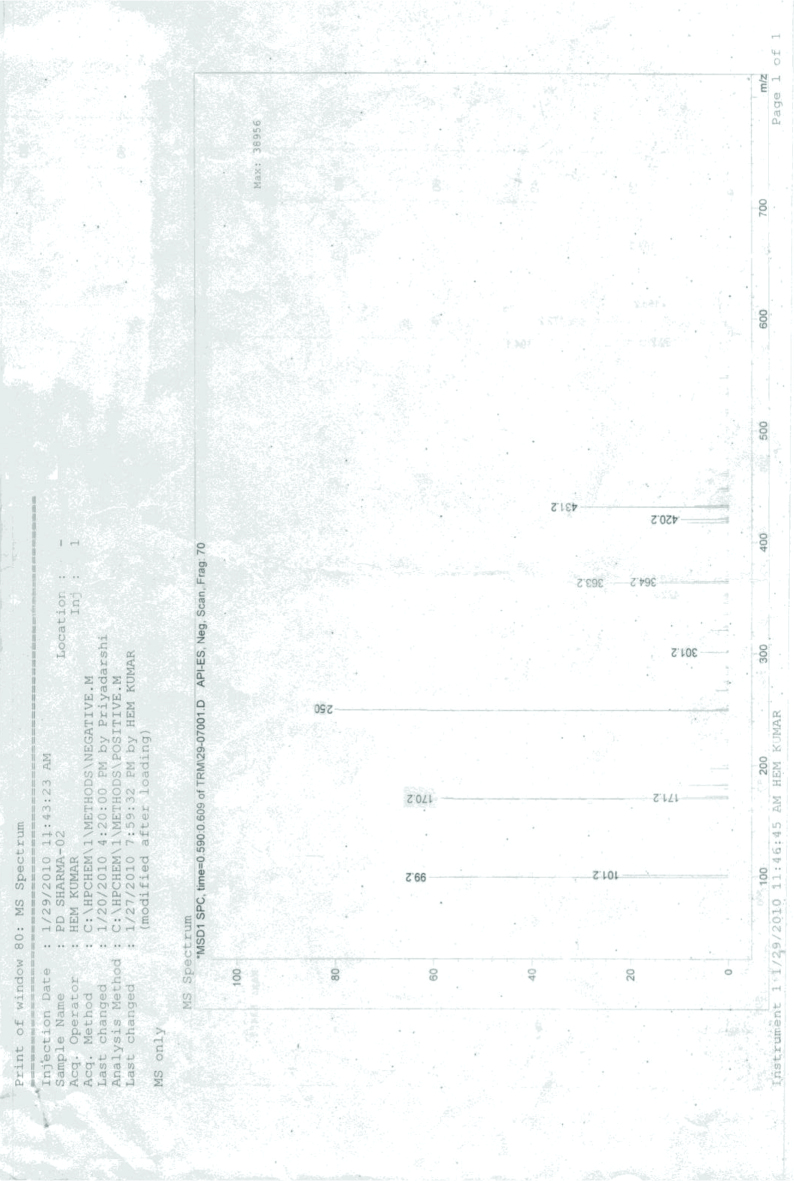
Mass spectra of 2,4-dinitrophenylhydrazone.

**Mass spectral analysis of 2, 4-dinitrophenylhydrazone** Mass spectra of 2,4-dinitrophenyl hydrazone of crotonaldehyde show molecular ion peak as base peak at 250 (Figure 16).

## 4. Conclusion

In uncatalysed reaction, the order is one with respect to the alcohol and oxidant each and rate is decelerated by hydrogen cation concentration. The kinetic evidence adequately substantiates a complex between Os(VIII), crotyl alcohol. The first order dependence with respect to chloramine-T suggests a ternary activated complex comprising oxidant, substrate and catalyst anion such an activated complex being bulky in nature ensues intramolecular hydrogen anion transfer from the alcohol to the oxidant which in turn further undergoes redox decomposition. It shows that electron deficient metal centre of the catalyst being coordinated through oxygen if crotyl alcohol exposes latter to the attack by the oxidant.
